# The alliance between genetic biobanks and patient organisations: the experience of the telethon network of genetic biobanks

**DOI:** 10.1186/s13023-016-0527-7

**Published:** 2016-10-24

**Authors:** Chiara Baldo, Lorena Casareto, Alessandra Renieri, Giuseppe Merla, Barbara Garavaglia, Stefano Goldwurm, Elena Pegoraro, Maurizio Moggio, Marina Mora, Luisa Politano, Luca Sangiorgi, Raffaella Mazzotti, Valeria Viotti, Ilaria Meloni, Maria Teresa Pellico, Chiara Barzaghi, Chiuhui Mary Wang, Lucia Monaco, Mirella Filocamo

**Affiliations:** 1 S.C. Laboratorio di Genetica Umana, E.O. Ospedali Galliera, Genoa, Italy; 2 Ufficio Coordinamento Network, c/o U.O.S.D. Centro di Diagnostica Genetica e Biochimica delle Malattie Metaboliche, Istituto G. Gaslini, Genoa, Italy; 3 Medical Genetics, University of Siena and Genetica Medica, Azienda Ospedaliera Universitaria Senese, Siena, Italy; 4 Medical Genetics Unit, IRCCS Casa Sollievo della Sofferenza, S. Giovanni Rotondo, FG Italy; 5 U.O.C. Neurogenetica Molecolare, Fondazione I.R.C.C.S. Istituto Neurologico Carlo Besta, Milan, Italy; 6 Parkinson Institute, ASST Centro Specialistico Ortopedico Traumatologico G. Pini – CTO, Milan, Italy; 7 Università di Padova, Azienda Ospedaliera Universitaria, Padova, Italy; 8 Neuromuscular and Rare Diseases Unit, Dino Ferrari Centre, IRCCS Foundation Ca’ Granda Ospedale Maggiore Policlinico, University of Milan, Milan, Italy; 9 Laboratorio di Biologia Cellulare, UO Malattie Neuromuscolari e Neuroimmunologia, Fond. IRCCS Istituto Neurologico Carlo Besta, Milan, Italy; 10 Cardiomiologia e Genetica Medica, Dipartimento di Medicina Sperimentale, Seconda Università di Napoli e Azienda Ospedaliera Universitaria della SUN, Naples, Italy; 11 S.S.D. Genetica Medica e Malattie Rare Ortopediche Istituto Ortopedico Rizzoli, Bologna, Italy; 12 U.O.S.D. Centro di Diagnostica Genetica e Biochimica delle Malattie Metaboliche, Istituto G. Gaslini, Via G. Gaslini 5, 16147 Genoa, Italy; 13 Fondazione Telethon, Milan, Italy

**Keywords:** Patient organisations, Rare diseases, Biobanking, Networking, Agreements, Sample and data centralisation, Patient involvement, Research

## Abstract

**Background:**

Rare diseases (RDs) are often neglected because they affect a small percentage of the population (6–8 %), which makes research and development of new therapies challenging processes. Easy access to high-quality samples and associated clinical data is therefore a key prerequisite for biomedical research. In this context, Genetic Biobanks are critical to developing basic, translational and clinical research on RDs. The Telethon Network of Genetic Biobanks (TNGB) is aware of the importance of biobanking as a service for patients and has started a dialogue with RD-Patient Organisations via promotion of dedicated meetings and round-tables, as well as by including their representatives on the TNGB Advisory Board. This has enabled the active involvement of POs in drafting biobank policies and procedures, including those concerning ethical issues. Here, we report on our experience with RD-Patient Organisations who have requested the services of existing biobanks belonging to TNGB and describe how these relationships were established, formalised and maintained.

**Results:**

The process of patient engagement has proven to be successful both for lay members, who increased their understanding of the complex processes of biobanking, and for professionals, who gained awareness of the needs and expectations of the people involved. This collaboration has resulted in a real interest on the part of Patient Organisations in the biobanking service, which has led to 13 written agreements designed to formalise this process. These agreements enabled the centralisation of rare genetic disease biospecimens and their related data, thus making them available to the scientific community.

**Conclusions:**

The TNGB experience has proven to be an example of good practice with regard to patient engagement in biobanking and may serve as a model of collaboration between disease-oriented Biobanks and Patient Organisations. Such collaboration serves to enhance awareness and trust and to encourage the scientific community to address research on RDs.

**Electronic supplementary material:**

The online version of this article (doi:10.1186/s13023-016-0527-7) contains supplementary material, which is available to authorized users.

## Background

The term rare diseases (RDs) refers to approximately 5000–8000 individual diseases that affect an estimated 350 to 400 million people worldwide. Because RDs affect a limited number of individuals (6–8 % of the population), they are often neglected. Therefore, research and development of new therapies are challenging processes that require considerable effort [1].

RDs have often a genetic origin and, although etiologically heterogeneous, they can share cellular and molecular pathways. Understanding their pathological and developmental bases may therefore lead to a new medical classification system and the development of shared therapeutic targets.

Given the nature of RDs, easy access to high-quality samples and associated clinical data is a key prerequisite for biomedical research. In this context, Genetic Biobanks (GBs), which are resources of samples and data that are collected, stored, processed, and distributed in an organised system, are critical to the progress of basic, translational and clinical research on RDs [2]. Therefore, close interaction between GBs and clinical/diagnostic centres is essential to maintaining well characterised samples linked to updated clinical data. Additionally, the involvement of RD Patient Organisations (POs) is essential if a critical mass of samples is to be obtained, as POs can serve as liaisons between GBs and physicians in collecting both samples and clinical data for research. A strong collaboration between GBs and POs is also fundamental to achieving the integration of sample data stored in biobanks and clinical data stored in registries or clinical databases. In this respect, the European FP7 program, RD-Connect, was designed to build an integrated platform that links registries to GBs and to clinical data for research on rare diseases [1, 3].

Over the last few years, the degree of involvement of POs in biobanking activities has increased greatly; POs have evolved from mere participants to active collaborators. This change is also due to the fact that patients, unhappy with the speed of research into their respective diseases, have started to look with interest at the opportunities provided by the biobanks. Individual patients and POs’ representatives are now part of the decision-making governance structure of national and international biobanks or biobank networks [4]. Here, they can express their points of view and expectations of research on their samples as well as participate in drafting ethical guidelines and operational procedures concerning sample use/transfer and data sharing. Within the rare disease patients’ community, there are also examples of those who have invested their own resources in the establishment of new own biobanks [4–6].

Here, we report on our experience with rare disease patient organisations that have requested the services (sample and data collection, preparation, storage and distribution) of existing biobanks belonging to the “Telethon Network of Genetic Biobanks” and describe how these relationships have been established, formalised and maintained.

## Methods

### ​Telethon Network of Genetic Biobanks

The Telethon Network of Genetic Biobanks (TNGB) [7], currently composed of 11 Italian non-profit repositories, was established in 2008 within the framework of a research project financially supported by the Telethon Foundation. Currently, TNGB stores more than 90,000 biological samples representing approximately 850 distinct rare genetic diseases. Responsibilities for harmonisation and standardisation – with regard to the collection, preparation, transport, storage, and distribution of samples – have been shared by all partners and are stated in the Network Charter.

One of the primary objectives of the project was to interconnect already well-established Italian GBs, most of which have been operating since the 1970s–1980s, through a unique and centrally coordinated IT infrastructure designed to (i) standardise and harmonise the procedures; (ii) minimise biases that might arise from heterogeneity in sample quality; (iii) develop a common sample access policy based on predefined criteria. Another central aim was to promote GB services within POs, with the goal of fostering their active participation and sharing benefits with them in terms of research findings [2]. To make this strategy practical, a representative of the RD Patient Organisation “UNIAMO FIMR” (an Italian Federation of approximately 100 Associations of patients with rare diseases) [8] was invited to join the TNGB Advisory Board and to contribute to the development of the TNGB. This arrangement has been in place since the conception of the TNGB.

The TNGB is not only a national reality. In fact, the 11 TNGB partners are also members of the EuroBioBank network, the first European network of GBs for rare diseases [6]. Moreover, since 2012, TNGB has also been an associated partner of the European RD-Connect project [9], which aims to connect databases, registries, biobanks and clinical bioinformatics for rare disease research [1, 3]. In addition, TNGB participates in the Biobanking and Biomolecular Resources Research Infrastructure [10].

### Engagement of Patient Organisations

The TNGB has begun a dialogue with the public, including patient organisations, by promoting several events (a total of 34 events over 9 years). This activity, publicised by an information leaflet, has helped to explain the purposes of biobanks and how they operate, as well as to give patients and families the opportunity to voice their perspectives, needs and concerns.

Furthermore, as we mentioned in the previous section, the participation of a PO representative on the TNGB Advisory Board has been an effective way for patients to be actively involved in drafting TNGB policies and in sharing their perspectives on procedures concerning ethical issues such as transparency, informed consent, privacy, sample use and transfer, data sharing, commercialisation, return of results and incidental findings [11–13].

Several activities occurring within the framework of the project “Determinazione rara”, financed by the Italian Ministry of Labour and Welfare and UNIAMO FIMR [14], have demonstrated additional examples of good practice with regard to patients’ engagement in biobanking. Through a series of organised roundtable sessions, the lay members of patient organisations and the panel of experts (biobankers, healthcare professionals, bioethicists, jurists/lawyers) discussed ethical and legal concerns related to privacy and informed consent, sample ownership, withdrawal of samples and consent, access to samples and data, return of results and incidental findings. This extremely fruitful debate led to a draft of comprehensive informed consent that became the official model adopted by all the biobanks in the Network [15].

### Agreements between TNGB and Patient Organisations

The interest of Patient Organisations in the biobank service led to the formalisation of a working partnership via a written agreement. An agreement template was drafted (and approved by the TNGB Advisory Board) with the aim of defining the terms and tasks of the parties viz. the interested PO and one of the 11 Biobanks of the Network. The selection of the Biobank is usually based on some pre-defined criteria, including geographical proximity to the PO’s head office and the affinity of the disease to the pre-existing collections.

**Fig. 1:**
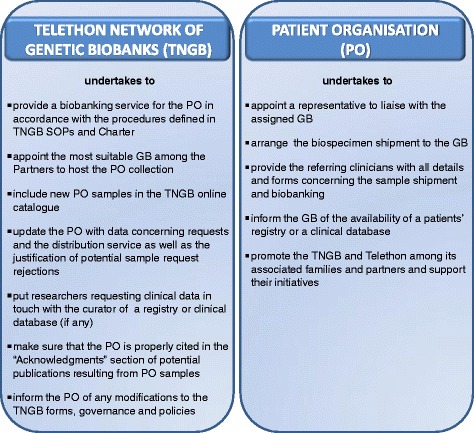
Tasks of the Parties

Briefly, the agreement template includes the purpose of the agreement, a detailed description of roles and responsibilities, a specification of the duration of the service and a clause on termination, and the parties’ signatures. Concerning the parties’ roles and responsibilities, the PO shall undertake to (i) identify a representative who keeps associated families and referring clinicians informed of the Biobank’s activities and policies; (ii) promote the recruitment of patients and relatives; and (iii) organise shipment of biospecimens to the assigned Biobank, which, in its role, undertakes to (i) provide the service of biobanking according to TNGB policies and SOPs; (ii) publish the sample collection on the TNGB online catalogue; and (iii) keep the PO’s representative informed of the sample workflow and availability of potential findings resulting from the distribution service (Fig. 1). The agreement template is available as Additional file 1. The parties sign the agreement for a period of one year, as a trial period. The trial serves to adapt the several procedures and to share forms already implemented by the Biobanks of the Network and, eventually, to decide whether the agreement should be renewed. In the event that the agreement is not renewed, the PO shall decide whether they wish to withdraw samples and related consents or transfer the samples to another Biobank. The agreement also includes, as annexes, the following forms: (i) subject and data sheet, tailored to clinical features of the disease to optimise data collection; (ii) informed consent form for seeking the subject’s consent to biobanking; (iii) material transfer agreement (MTA) template, made available for the PO’s consultation. Indeed, while the informed consent model was drafted through an iterative process involving the patients (as described above), the MTA template, as a legal instrument that defines terms for the transfer of biological materials between two Institutions [16], was drafted by the Telethon Technology Transfer Office and subsequently discussed during a meeting of the TNGB Advisory Board, which included a representative of the RD Patient Organisation.

Once the agreement is signed, the Biobank staff work in close collaboration with the PO’s representative and the panel of referred clinicians to define the sample type(s) to be collected and the operative procedures related to their sampling and shipment. Sample collections from patients occur in various ways and are generally organised to coincide with the PO’s biannual/annual meetings (which update members on the latest research and developments concerning their diseases) or during open days, which include a systematic multidisciplinary assessment of the patients. To support the PO, the director of the involved GB always attends the meetings to (i) provide further details concerning the terms of the agreement; (ii) emphasise the importance of making genuine and conscious choices before consenting to biobanking; (iii) address potential questions and concerns during the process of seeking informed consent; (iv) give further technical details about data sharing practices through the use of the legal instrument (MTA); (v) emphasise that the potential benefits derived from the use of the samples can impact not only the health of the individual but also the entire community in accordance with the concept of the “common heritage” in relation to the human genome, invoked by UNESCO and HUGO [17–19]; (vi) update the PO about samples and data workflow, possible research results and publications obtained from the sample collection.

To increase the visibility of the samples collected under the agreements and to spread knowledge of the sustained engagement and involvement of patients in biobanks, a web page has been created on the TNGB web site that is entirely dedicated to activities with POs [20].

## Results

Events and meetings have been very effective in maintaining patients’ interactions with professionals and thus increasing their literacy and empowerment. This two-way information exchange has allowed the professionals, on the one hand, to provide information that helps patients understand (i) the length of the diagnosis and research processes, (ii) the measures for sharing data with researchers requesting samples, (iii) the procedures for the return of results, and (iv) the international and national recommendations and norms regulating biobanking. The patients, on the other hand, have had the opportunity to raise the awareness of the professionals by (i) providing information on their values, priorities, needs, perspectives and expectations concerning the biobank services; and (ii) sharing their points of view on ethical issues with regard to informed consent, data sharing and return of findings. From the several debates that occurred, it became clear that the concerns and reservations – expressed by both patients and the public – are not particularly related to privacy and data sharing issues but rather to the fact that the use of their samples is often limited to the research centres that collected the samples. Another critical issue concerns the difficulty of obtaining access to the results of research performed using their samples and data. The issue of the mode of returning results, including incidental ones, has indeed generated a broad debate with the patients. Based on the general ethical principle that patients have the right to decide if they want to know or not know the results of the research, the central question is who should communicate the results and which results should be returned, as they can differ in validity, predictive power and clinical significance [21]. Concerning this last point, we have helped patients understand the objective difficulty of communicating intermediate research results (that do not have immediate applicability) and, in addition, disclosed that while the Biobank staff must do everything within its power to ensure the return of research results, they may not have the specific expertise required to communicate such results. In this event, the Biobank staff should be acting as a link between the researchers and the people who are best suited to contact the patients, such as the referring clinician together with the geneticist. This option has been introduced in the TNGB form seeking patients’ consent, thus inviting patients to indicate their preferred referral centre.

### Agreements between Patient Organisations and the Genetic Biobanks of the Network

**Fig. 2:**
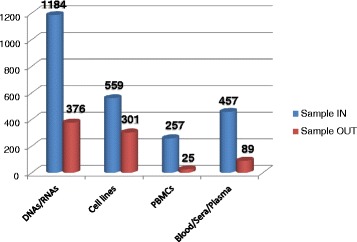
Sample workflow per type in the framework of the agreement Legend: Sample IN = incoming samples; Sample OUT = outgoing samples; PBMCs = Peripheral blood mononuclear cells; cell lines include fibroblasts, Epstein-Barr virus lymphoblasts, Induced Pluripotent Stem Cells, amniocytes and trophoblast cells

The various events mentioned above have increased awareness, trust and interest in the biobank service on the part of Patient Organisations and, as of now, have led to 13 agreements. The first was signed in December 2009 between the Ring 14 Italian Association and the Galliera Genetic Bank, based on the biobank staff’s experience in the diagnosis of chromosomal disorders [22]. Since then, another 12 POs have expressed their interest in TNGB services and formalised agreements that involved five different TNGB partners (Table 1). The 13 agreements have allowed TNGB to centralise very rare samples in a unique catalogue: as of now, TNGB has collected 2457 samples derived from 616 affected subjects and 518 relatives (Table 1). This result has been achieved thanks, in part, to tailored procedures and forms for collecting samples and associated data, developed together with PO representatives and referring clinicians. [23]. Concerning the outcomes derived from the online visibility of these disease collections, 52 sample requests from the international scientific community have been processed, leading to the distribution of 791 biospecimens. Figure 2 shows the workflow of samples in terms of quantity and typology.

**Table 1:** Summary information concerning the agreements currently in place

Genetic Biobank	Patient Organisation	Disease	Starting date	Patient Registry/Clinical database	No. of subjects
Cell line and DNA Biobank from patients affected by genetic diseases (TNGB Coordinator)	Associazione Italiana Sindrome di Poland	Poland S. (ORPHA2911)	Feb 2014	Clinical database	238
	LND Famiglie Italiane	Lesch-Nyhan disease (ORPHA510)	Oct 2014	Italian Patient Registry (in progress)	6
	F.O.P. Italia Onlus	Fibrodysplasia ossificans progressive (ORPHA337)	Jan 2015	No	4
Galliera Genetic Bank (TNGB Partner 1)	Ring 14 International	Ring 14 S. and other chromosome 14 related diseases (ORPHA1440)	Dec 2009	Clinical database	197
	Associazione Nonsolo15	dup15q S. (ORPHA3306)	Jul 2012	No	40
	Associazione Mowat Wilson	Mowat-Wilson S. (ORPHA2152)	Jul 2012	No	27
	Gruppo Famiglie Dravet	Dravet S. (ORPHA33069)	May 2013	Clinical database	56
	ASSI Gulliver - Sindrome di Sotos	Sotos S. (ORPHA821)	Jul 2015	No	4
Cell lines and DNA Bank of Rett syndrome, X-linked mental retardation and other genetic diseases (TNGB Partner 3)	Associazione Sindrome di Alport	Alport S. (ORPHA63)	Oct 2013	Italian Patient Registry	247
	Associazione Italiana Rett	Rett S. (ORPHA778)	Nov 2013	Italian and European Patient Registry	95
	ILA - Associazione italiana Angiodisplasie ed Emangiomi Infantili	Vascular Malformations	May 2014	No	24
Genomic and Genetic Disorders Biobank (TNGB Partner 7)	Federazione Italiana Prader Willi	Prader Willi S. (ORPHA739)	Jul 2012	No	124
Cell line and DNA Bank of genetic movement disorders and mitochondrial diseases (TNGB Partner 9)	AISNAF - Associazione Italiana Sindromi Neurodegenerative da Accumulo di Ferro	Neurodegeneration with brain iron accumulation (ORPHA385)	May 2015	No	72

*TNGB* Telethon Network of Genetic Biobanks, *S* Syndrome

In line with the mission of the TNGB, the significant increase in the variety of incoming and outgoing samples reflects the Biobanks’ efforts to satisfy the current and future demands of the researchers engaged in studies of these rare diseases.

All samples and data collected under the agreements were made available to the scientific community on the basis of the access rules stated in the Network Charter and shared with the PO’s representatives; this ensures transparency and privacy protection throughout all phases of the process. Three scientific papers (acknowledging both the GB and the PO) resulting from the use of the distribution service have been published [24–26].

The “Ring 14” collection is the best example of how this model of cooperation has worked efficiently in terms of both number of subjects recruited and their geographical provenance. This result has been achieved thanks to the recent creation of the second level association “Ring 14 International”, which has greatly facilitated the centralisation of samples and data of subjects with different geographical origins (i.e., Italy, Europe, USA) into a single biobank and a clinical database [27]. Indeed, the families, being consciously involved in the collection process, have felt themselves to be key players in fostering research on their neglected disease. The availability of a centralised collection of a critical mass of these extremely rare samples has stimulated the researchers’ interest and prompted some POs (i.e., Ring 14 International, Gruppo Famiglie Dravet, AIRETT-Associazione Italiana Rett) to financially support specific research projects selected through a peer-review process. This involvement allows the POs to play an active role in combating the disease from which they suffer [28].

Finally, the close ties between POs and GBs also facilitate the sharing of related clinical data and ongoing work on integrating different RD resources in line with the primary aim of RD-Connect.

## Discussion

The process of public involvement in biobanks proved to be successful for both the lay members and the professionals involved. The public has been able to increase their understanding of the complex processes of biobanking and has become more confident. At the same time, professionals have gained knowledge of the needs and expectations of the people involved and learnt to be much more open to discussion concerning ethical issues. The results of the first six years of activities within the framework of these agreements have provided evidence that POs’ collaboration is instrumental in centralising both rare samples and associated data, as well as giving visibility to these collections through the TNGB online catalogue, which has captured the interest of international researchers studying neglected diseases. Another advantage of this approach is that POs have been directly involved in research advancement by playing an active role in the mechanisms of RD research and also by contributing with seed grants to support research projects focused on their diseases.

Another positive aspect of this collaboration can be seen in the increased effort POs have put into sharing their knowledge of biobanking with affiliated families and with society as a whole to improve awareness of patients’ rights and to address critical issues such as privacy protection, use of samples and data, and return of research findings to patients. This activity has enabled POs to actively participate in drafting transparent regulations and guidelines for sample management as well as contribute to the alignment of some TNGB procedures and forms with the needs of patients, such as the renewed informed consent form, drafted within the framework of the “Determinazione Rara” project, as well as procedures tailored to the samples and data collected under the agreements.

Finally, the TNGB-PO’s agreements demonstrate how active collaboration between GBs and POs is critical to rare disease biobanking.

## Conclusions

To the best of our knowledge, this type of agreement is unique at the national and international level. The set of rules and tasks of the parties indeed ensures (i) quality and proper use of the samples, (ii) individuals’ confidentiality throughout the entire process and, more importantly, (iii) visibility of and easy access to a specific sample collection for the interested biomedical community.

The TNGB experience may therefore serve as a model of collaboration between disease-oriented Biobanks and Patient Organisations, as it shows how mutual respect and effective collaboration between patients and the scientific community are essential to the enhancement of awareness and trust, as well as to the sharing of objectives and efforts to support research on rare diseases.

## Additional file

Additional file 1: Agreement template. (PDF 217 kb)

## Abbreviations

BBMRI: Biobanking and Biomolecular Resources Research Infrastructure
FP7: Seventh Framework Programme for research and technological development
GB: Genetic Biobank
HUGO: Human Genome Organisation
MTA: Material Transfer Agreement
PO: Patient Organisation
RD: Rare Disease
TNGB: Telethon Network of Genetic Biobanks
UNESCO: United Nations Educational, Scientific and Cultural Organization
