# Supplementary material for: The alliance between genetic biobanks and patient organisations: the experience of the telethon network of genetic biobanks
Source: Orphanet J Rare Dis. 2016 Oct 24;11:142. doi: 10.1186/s13023-016-0527-7 (PMC5078978; doi:10.1186/s13023-016-0527-7)
Supplement: Additional file 1: — Agreement template. (PDF 217 kb) [file 13023_2016_527_MOESM1_ESM.pdf]

TELETHON NETWORK OF GENETIC BIOBANKS, Project n. GTB12001
Email biobanknetwork@tellethon.it
Web http://biobanknetwork.telethon.it/

Patient Organisation Logo

[Patient Organisation Name]
[Patient Organisation Address]
[Patient Organisation contacts and URL]

Agreement for the management of the collection of biological materials from individuals affiliated to *[Patient Organisation Name]*

Preamble:
Telethon Foundation ("TF") is a non-profit organization supporting medical and scientific research on neuromuscular and other genetic diseases conducted by Organizations, Institutions and Associations with the same aim.
In 2008, TF established the Telethon Network of Genetic Biobanks ("TNGB", http://biobanknetwork.telethon.it/) to coordinate by using an IT infrastructure, a number of Italian Genetic Biobanks, which are financially supported by TF and evaluated by the Telethon Biobank Committee through a peer-review selection. TNGB's objective is to offer its services to Patients and Patient Organisations by centralising collections of rare genetic disease biospecimens and their related data, as well as to make them available to the Scientific Community and to families.
TNGB is the first Italian network to share Standard Operating Procedures (SOPs) and guidelines on legal, ethical and quality related issues in compliance with national and international laws and recommendations. The TNGB Charter defines these issues as well as the relationships between TNGB partners.

[1-year preliminary agreement] The *[Patient Organisation Name]* is interested in using TNGB services.

[Renewal] The *[Patient Organisation Name]* and TNGB signed an agreement on *[date of the signature]* for management of biological material samples from individual members. The *[Patient Organisation Name]* (hereinafter "Patient Organisation") wishes to continue using TNGB services.

Both TNGB and the Patient Organisation's Charters are an integral part of this agreement and are mutually accepted.

Parties:
1. *[Patient Organisation Name]*, duly represented by *[Name]*, President
2. *[Biobank Name]*, duly represented by *[Name]*, Biobank Director, (hereinafter "Biobank") and TNGB, duly represented by *[Name]*, TNGB Coordinator, on behalf of TF (TNGB founding organisation) having on *[Date]* received the authorisation from FT to sign this agreement.

Aims:
The aim of this agreement is to define the rules and tasks of the parties involved, for storage and distribution of biospecimens and related data from the individual members and families affected by *[Disease of interest of the Patient Organisation]*.

Tasks of the Parties

[Patient Organisation Name] undertakes to:
- promote the biospecimen transfer to the *[Biobank Name]* (a TNGB partner), located in *[Biobank Address]*;
- inform the referring clinicians responsible for taking samples as well as the laboratory responsible for the sample collection and preparation concerning documentation (informed consent and sample submission forms) needed for sample transfer and storage in the Biobank;
- select a representative of the Patient Organisation to liaise with the Biobank. The Patient Organisation can substitute its representative at any time;
- support the referring clinician / centre in organising sample taking and transfer to the Biobank;
- if applicable, inform the Biobank that a registry or clinical database containing data related to the stored samples is available;
- promote TNGB and TF among its associated families and partners and support their initiatives.

TNGB undertakes to:
- provide a service of sample storage and distribution for the Patient Organisation in accordance with the procedures defined in TNGB SOPs and Charter;

- appoint the Biobank as entity hosting "*[Name of the collection]*" collection by agreement with TF and the Patient Organisation;
- include the sample collection into the TNGB online catalogue;
- keep the Patient Organisation updated about the sample workflow;
- inform the Patient Organisation about any sample request rejection, explaining the reasons for rejection;
- inform the Patient Organisation at the earliest possible time about any possible closures/relocations of the Biobank or about any alterations to the TNGB Charter or organisational changes which may affect this agreement or modify its terms;
- inform the Patient Organisation about any modifications to annexes (informed consent, sample submission, sample request forms). Revised versions of these annexes will replace the ones previously approved;
- inform the researchers requesting the "*[Name of the collection]*" clinical data about the existence of a clinical database or of a registry (if any);
- ask researchers accessing the "*[Name of the collection]*" biospecimens to (i) cite the Patient Organisation in the "Acknowledgments" section of possible publications and (ii) send a copy of the publication to the Patient Organisation.

<u>Duration</u>:
[1-year preliminary agreement] This Agreement is valid for 12 months from the date of the signatures by the Parties. At the end of this period, if both parties are satisfied, this agreement can be renewed in writing for an agreed period, no later than the end of the TNGB project financing termed as the *[Date]*.

[Renewal] This Agreement is valid until the end of the TNGB project financing termed as the *[Date]*. At the end of this period, if both parties are satisfied, this agreement can be renewed in writing for an agreed period if TNGB project financing is renewed.

If they deem it necessary, either parties can at any time and for any reason withdraw from this agreement with 3 months written notice, which is to be sent by registered mail or by PEC (Certified Electronic Mail). In this case, biospecimens should be returned to the Patient Organisation who, in agreement with TF and TNGB, could decide to transfer them to another TNGB partner.

<u>Express termination clause:</u>
This agreement will terminate by rights under the Ex Article 1456 of the Italian Civil Code in the following cases:
i) proven non-fulfilment of the clauses of this agreement;
ii) ceased activity of one of the parties;
iii) amendment to Parties' Charters that are considered in contrast with principals and procedures stated in this agreement and/or not agreed/communicated.

The following annexes, approved by all Parties, form an integral part of this agreement:
- Informed consent to the storage of biological material (ANX. 1.A)
- Sample submission form (ANX. 1.B)
- Material transfer agreement form (ANX. 1.C)

Date

Seen and approved by

[Name]	*[Name]*	*[Name]*
TNGB Coordinator	Biobank Director	*[Role]* of *[Patient Organisation Name]*
______________________	______________________	______________________
